# Supplementary figures and images for: Emotion brain network topology in healthy subjects following passive listening to different auditory stimuli
Source: PeerJ. 2024 Jul 19;12:e17721. doi: 10.7717/peerj.17721 (PMC11262303; doi:10.7717/peerj.17721)

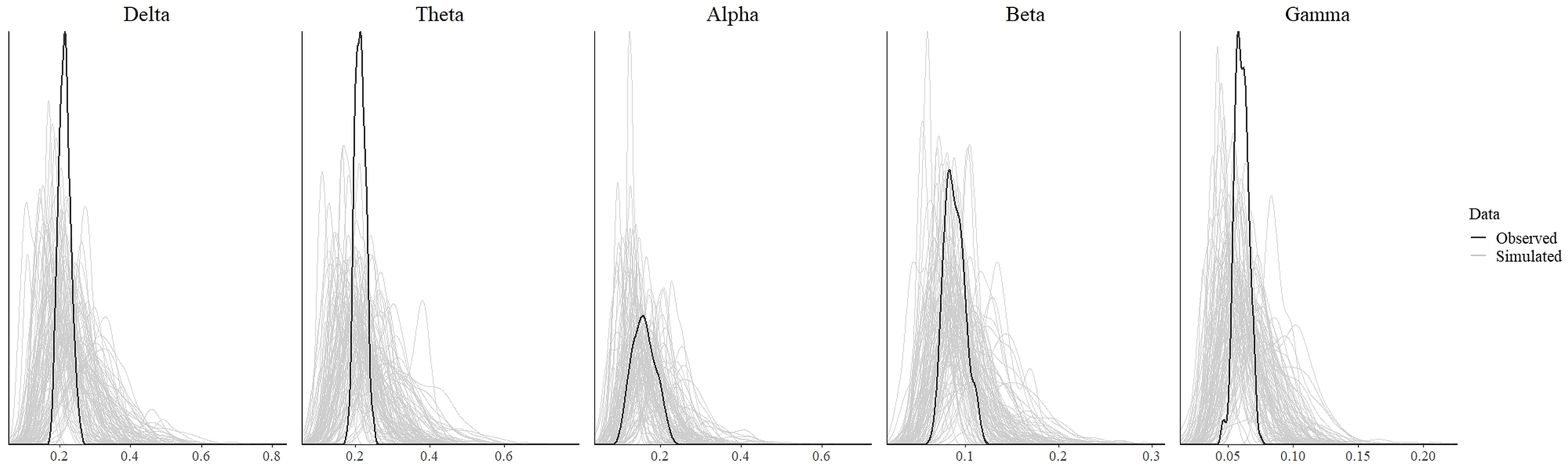

Supplement: Supplemental Information 1 [file peerj-12-17721-s001.jpg]

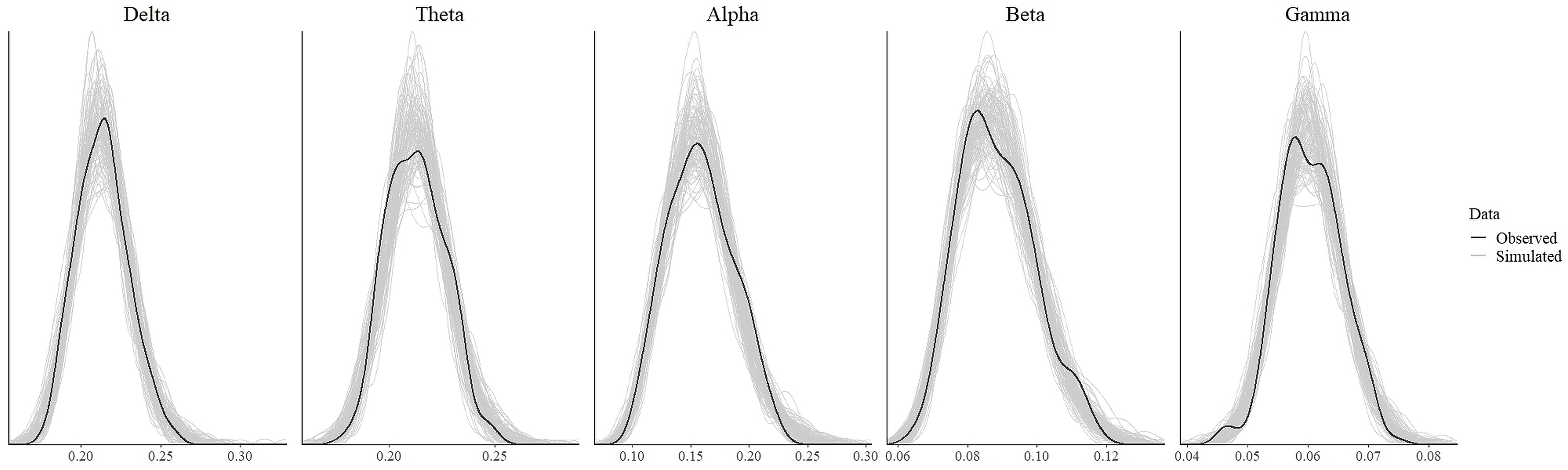

Supplement: Supplemental Information 2 [file peerj-12-17721-s002.jpg]

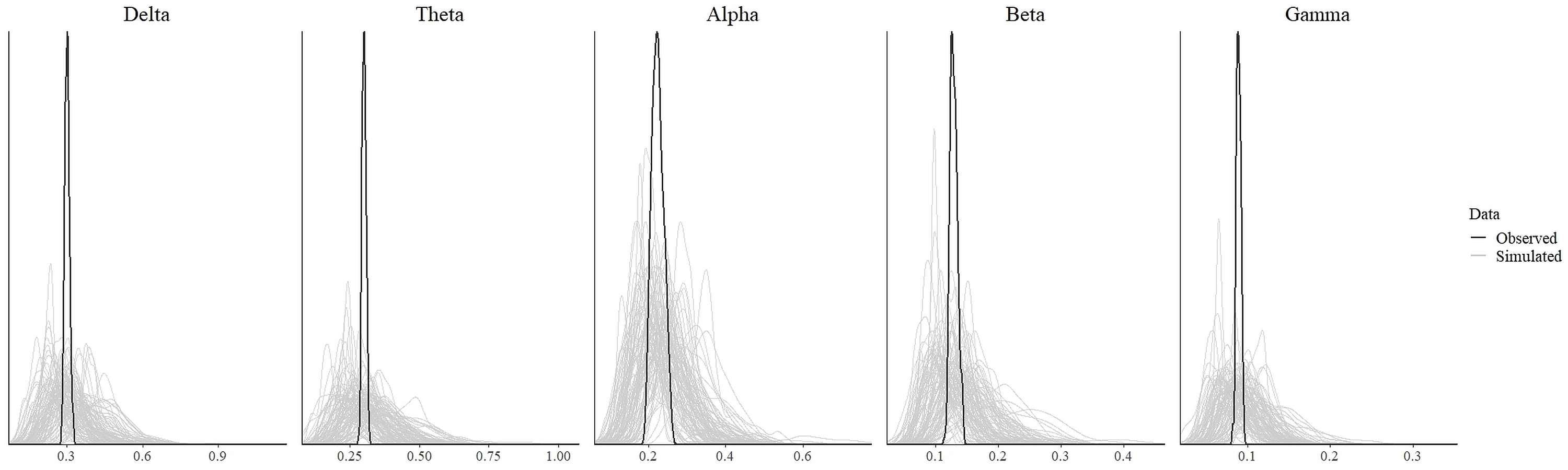

Supplement: Supplemental Information 3 [file peerj-12-17721-s003.jpg]

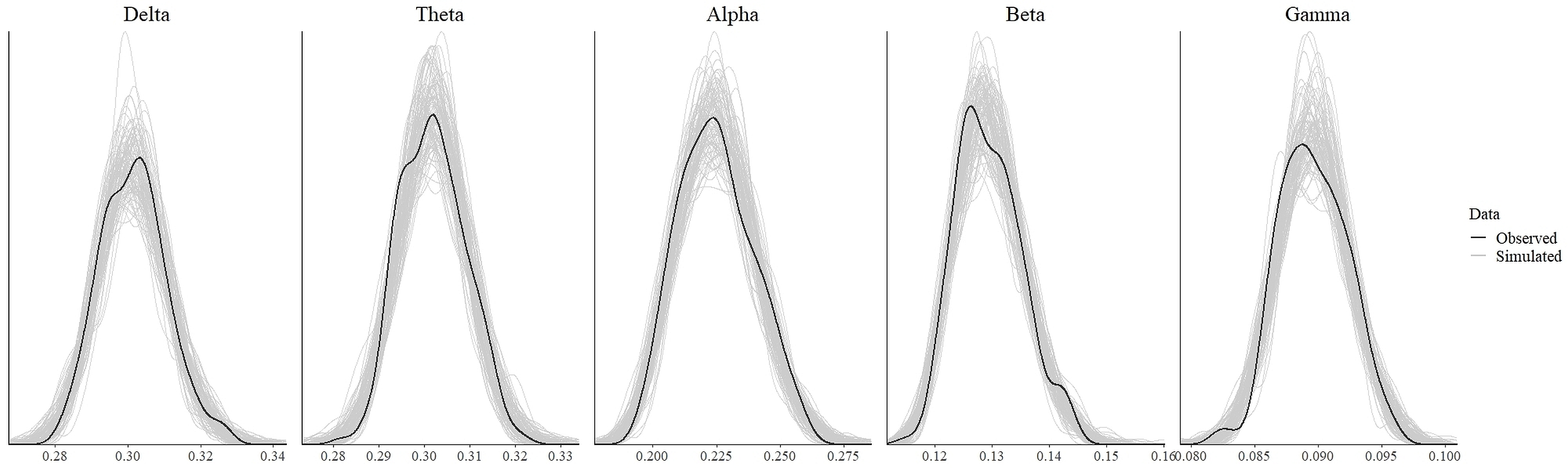

Supplement: Supplemental Information 4 [file peerj-12-17721-s004.jpg]

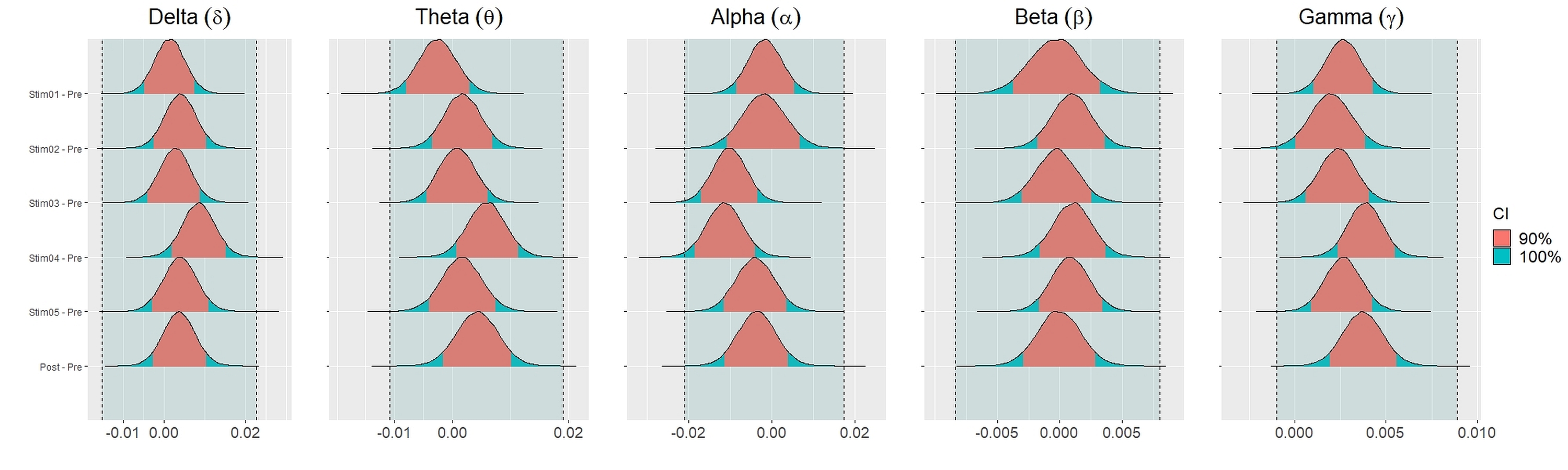

Supplement: Supplemental Information 5 [file peerj-12-17721-s005.jpg]

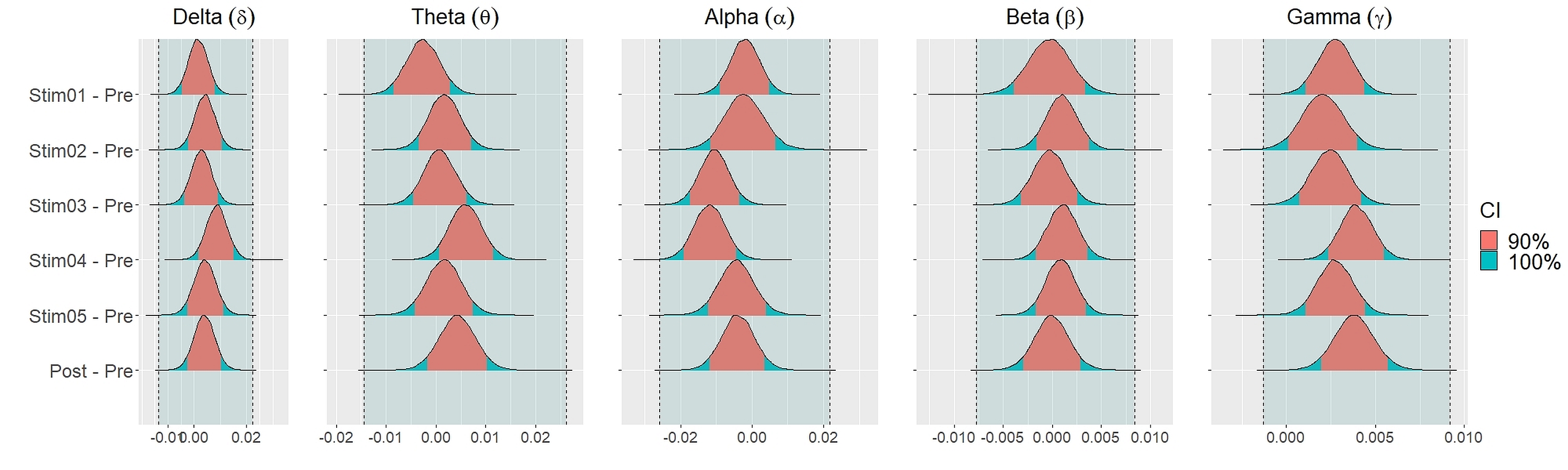

Supplement: Supplemental Information 6 [file peerj-12-17721-s006.jpg]

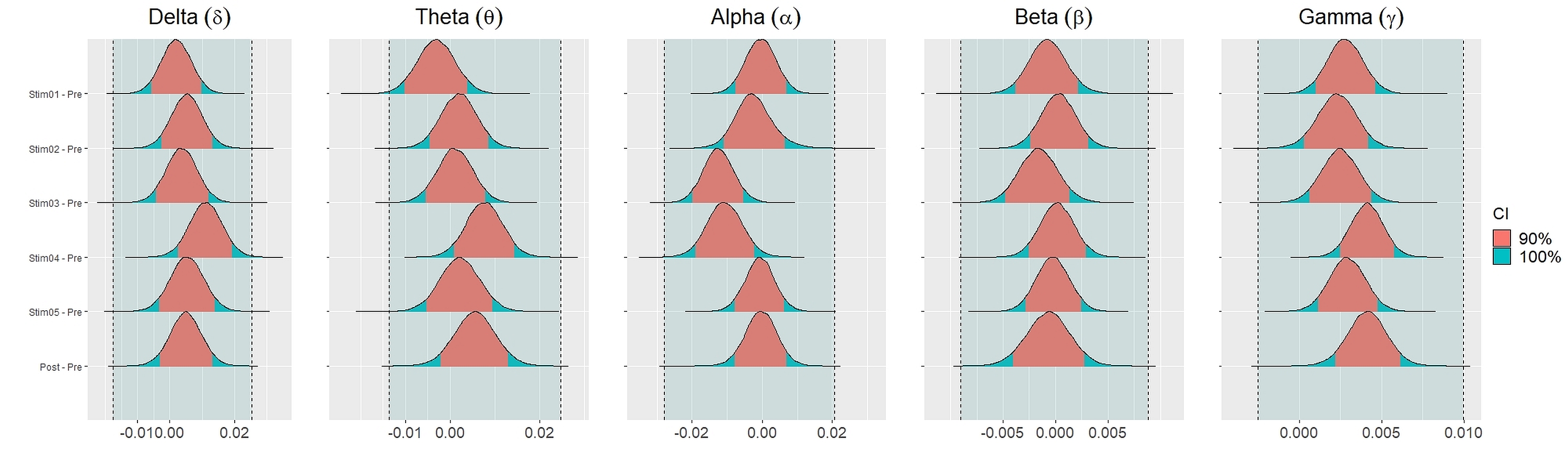

Supplement: Supplemental Information 7 [file peerj-12-17721-s007.jpg]

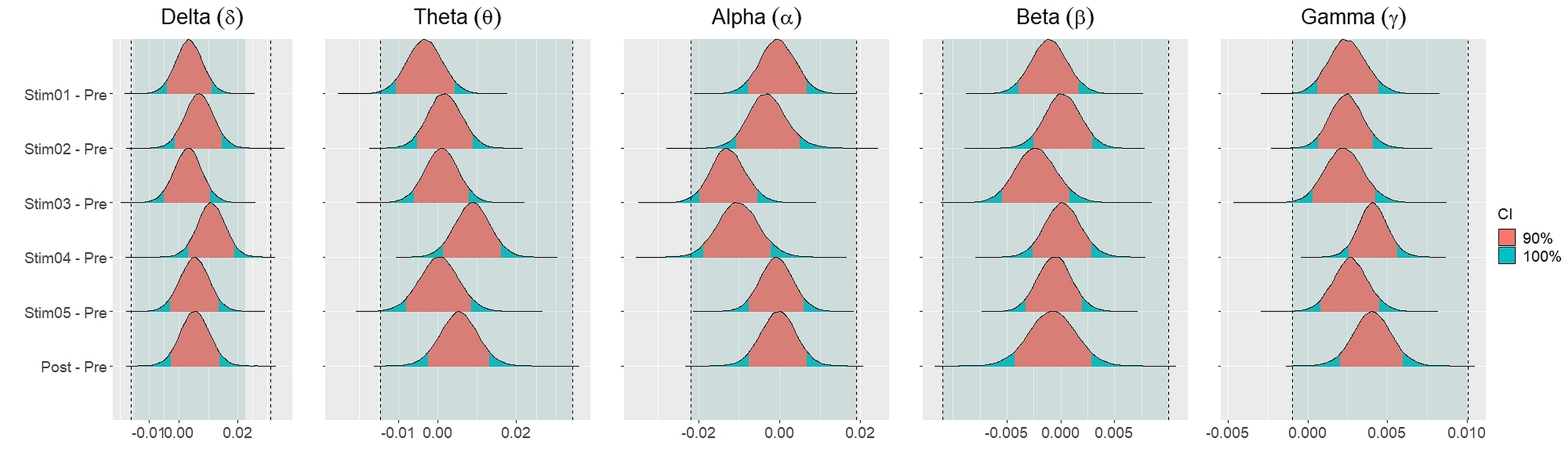

Supplement: Supplemental Information 8 [file peerj-12-17721-s008.jpg]

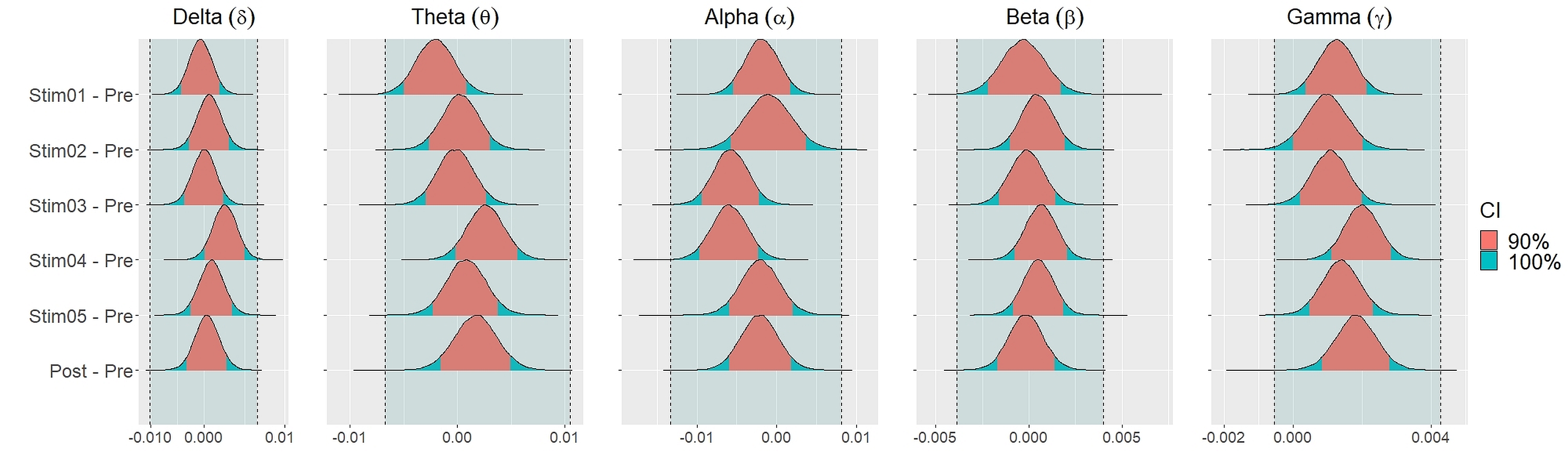

Supplement: Supplemental Information 9 [file peerj-12-17721-s009.jpg]

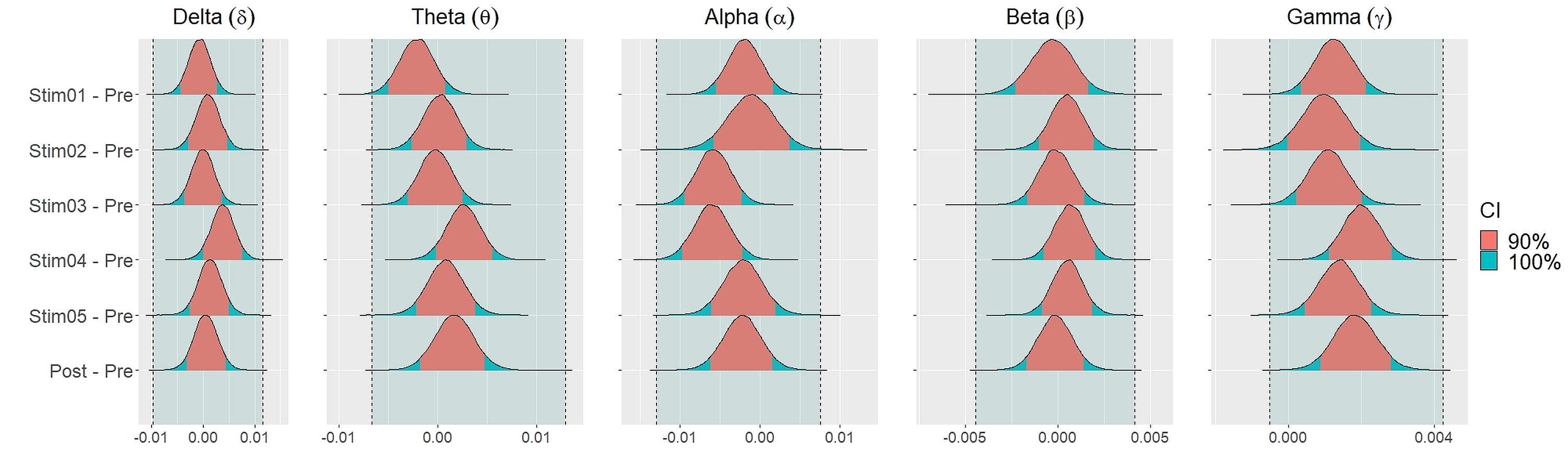

Supplement: Supplemental Information 10 [file peerj-12-17721-s010.jpg]

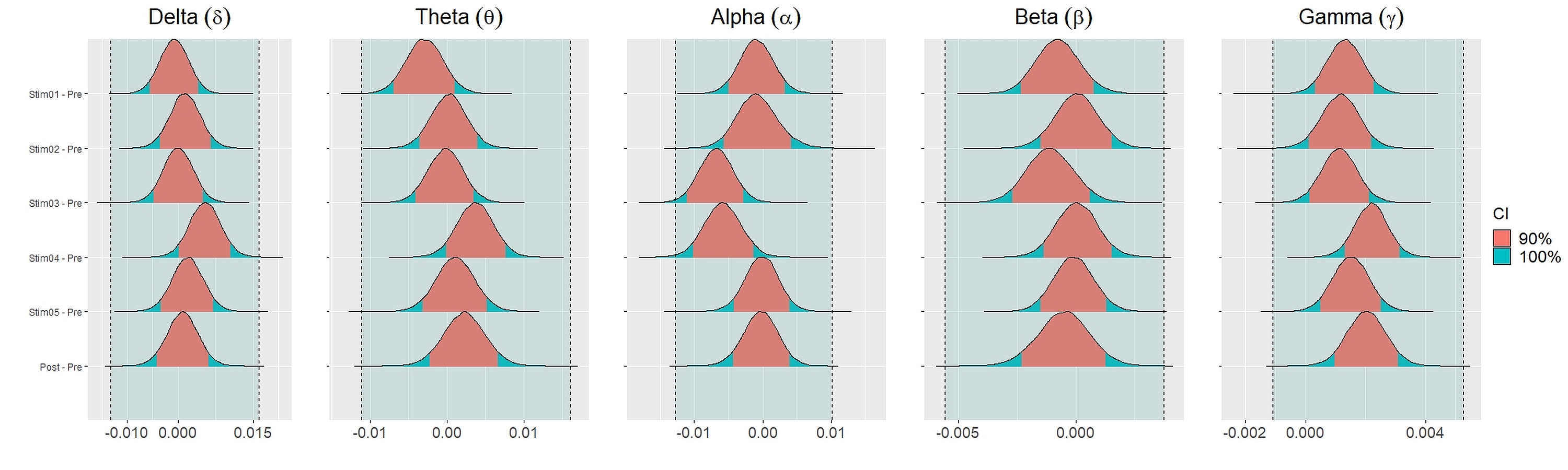

Supplement: Supplemental Information 11 [file peerj-12-17721-s011.jpg]

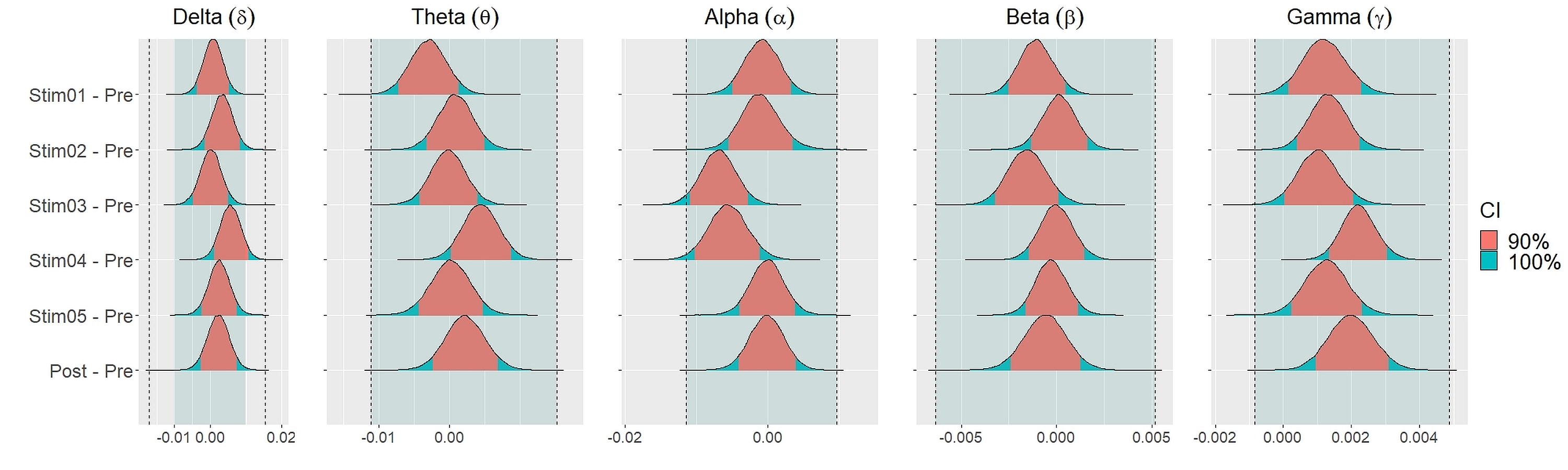

Supplement: Supplemental Information 12 [file peerj-12-17721-s012.jpg]
